# Rewilding the upper Klamath River Basin: rapid recolonization of Chinook salmon following the world’s largest dam removal

**DOI:** 10.1038/s41598-026-65437-0

**Published:** 2026-08-27

**Authors:** Damon H. Goodman, Keith Denton, George R. Pess, James Whelan, Oleksandr Stefankiv, Toz Soto, Alex Corum, Jordan Ortega, Oshun O’Rourke, Mark E. Hereford, Domenic Giudice, Nicholas A. Som

**Affiliations:** 1 California Trout, San Francisco, CA USA; 2 Keith Denton and Associates, Sequim, WA USA; 3 George Pess Consulting, Seattle, WA USA; 4 Karuk Tribe, Orleans, CA USA; 5 Klamath Tribes, Chiloquin, OR USA; 6 Yurok Tribe, Klamath, CA USA; 7 Oregon Department of Fish and Wildlife, Klamath Falls, OR USA; 8 California Department of Fish and Wildlife, Yreka, CA USA; 9 U.S. Geological Survey California Cooperative Fish and Wildlife Research Unit, California State Polytechnic University, Humboldt, Arcata, CA USA

**Keywords:** Ecology, Ecology, Environmental sciences

## Abstract

Habitat fragmentation is a global driver of decline in biodiversity, yet evaluations of ecological response following re-connection remain rare. In 2024, four hydroelectric dams on the Klamath River were removed, reconnecting over 640 km of anadromous habitat and marking the largest dam removal project completed to date. We used a stationary high-resolution multi-beam imaging SONAR to quantify adult Chinook salmon (*Oncorhynchus tshawytscha*) passage into newly reopened reaches during the first two migratory seasons following dam removal. An estimated 7,742 (95% CI: 7,702–7,778) Chinook salmon migrated upstream in 2024 and 13,310 (95% CI: 12,876–13,733) in 2025, representing 18–19% of fish returning to the Klamath River Basin. Within this period, Chinook salmon recolonized 88% of their documented historical distribution, expanding into 345 km of reconnected habitats and reaching elevations of up to 1,250 m. Recolonization required fish to swim through dam removal sites, complex hydraulic corridors and engineered fishways, demonstrating the propensity for rapid recolonization at basin scale despite these challenges. These findings provide the first quantitative assessment of salmon response to the world’s largest dam removal and demonstrate how reconnection can catalyze large-scale recolonization in historically fragmented river networks.

**Supplementary Information:**

The online version contains supplementary material available at https://doi.org/10.1038/s41598-026-65437-0.

## Introduction

Habitat fragmentation and associated impacts are primary drivers of global biodiversity decline and ecosystem dysfunction^1^. Fragmentation can cause the non-random loss of species important for ecosystem function, or restrict individual movement among sites, limiting the adaptive capacity of populations to climate vulnerability^1,2^. Impacts of habitat fragmentation can extend to human communities by reducing the benefits of ecosystem services^3^. Reconnecting fragmented habitats is a widely applied strategy to enhance ecosystem resilience, restore species, and support human well-being^4–6^. Demonstrated benefits of large scale reconnection projects are emerging across terrestrial, marine, and freshwater ecosystems with evidence of recovery as migratory behavior resumes, animals expand their distributions and lost ecosystem processes re-establish^7–9^. Reestablished animals can change the trophic structure of ecosystems, allow for range expansion, and promote long-term species population recovery^6,10,11^. For example, protection, range expansion, and increased connection between important habitats in Mozambique’s Parque Nacional da Gorongosa has helped to increase large-mammal biomass since war ended in 2004^12^. Connectivity is now recognized as foundational to restoration in marine ecosystems^13^. Large-scale reconnection projects remain rare – hindering our ability to learn from and predict ecological outcomes in fragmented ecosystems^7^.

River networks provide a framework for reconnecting ecosystems via their linear topology and the discrete nature of anthropogenic barriers^14^. The connectivity of river networks is vital to human wellbeing, ecosystem resilience, and biological diversity^14–16^. River connectivity is the basis for restoring and recovering river systems and associated freshwater populations of animals including salmonids^14,17,18^. If connectivity is lost due to a barrier or set of barriers, restoring connectivity then allows for re-expression of ecological processes and functions, and provides a mechanism for ecological, economic, and culturally important taxa such as salmonids to expand their range, resulting in increased life history diversity and larger populations^17^.

Dam removal is a tool to restore river connectivity, making it an ideal real-world experiment for restoring and evaluating river connectivity benefits. Dam removal also allows for the quantification of ecosystem response^16,19–22^. Dam removal restores longitudinal connectivity, resulting in a more natural hydrologic regime, continuous movement of sediment, seasonally variable fluxes of wood, nutrients, and energy^23,24^. Dam removal can reshape ecological communities by enabling the re-establishment of native species and, in some cases, altering conditions in ways that reduce prevalence of invasive species^20,25^. River ecosystem assemblages following dam removal may be very different than those before dam construction^19^. One focus of reconnecting river networks has been the re-establishment of migratory fishes above former dams, which can help develop native assemblages throughout a river ecosystem^9,19^. Migratory fishes can respond quickly to dam removal with increases in density, biomass, diversity, and abundance of natural fish assemblages above former barriers^19,26,27^. Linking habitats such as tributaries, large mainstem rivers, estuaries, and marine environments allows for the re-expression of life history strategies having been muted for decades to centuries^15,19,28,29^. The expression of diverse life history strategies is foundational to the long-term resilience of migratory fish populations^30–32^.

The Klamath River, stretching over 400 km from southern Oregon to the northern California coast, is the ninth largest river basin on the North American west coast. Its watershed spans 40,000 km^2^, encompassing a diverse range of habitats including high desert, spring creeks, alpine lakes, coniferous forests, and coastal estuaries^33^. The Klamath River Basin supports a diverse and endemic fish fauna with over 30 species of native fish^33–35^. Among these, Chinook salmon (*Oncorhynchus tshawytscha*) play a central role in the river’s ecological integrity, cultural heritage, and economy^36^. Salmon contribute essential nutrients to both aquatic and terrestrial ecosystems during their spawning runs^37^. The Klamath salmon runs are also economic drivers of the region supporting tribal subsistence harvesting, as well as sport and commercial fisheries^38^. For tribal nations that reside within the Klamath watershed, salmon are integral to traditional ways of life, ceremonies, and food security^38^.

Dams have restricted the distribution of fish in the Klamath River for over a century^39,40^. The dams, in addition to historical over-harvest, land-use effects, and water withdrawals have reduced the population size of migratory fishes by over 90%^34^. Disease has also played a substantial role in the reduction of Klamath River salmon populations^41,42^. Klamath River salmon have experienced chronically high infection and mortality from parasites, with outbreak severity strongly linked to dam-altered hydrology, warm temperatures, and low flows, culminating in 2002 when over 33,000 adult Chinook salmon, a quarter of the run of the Klamath River, died before reproducing, marking the largest recorded fish kill in the western United States^41^. Loss of connectivity to upstream cold-water habitats reduced genetic and life history diversity, with a loss of the spring-run Chinook salmon ecotype that was historically abundant and reliant upon spring-fed summer-holding habitats^43–45^. The decline of the river’s salmon populations helped coalesce a movement to remove four upper river hydroelectric dams^34^. This monumental effort culminated on 2-Oct-2024 when deconstruction of the four dams was completed. The four dams were J.C. Boyle (RKM 366.9, 18 m high, constructed in 1958), Copco 1 (RKM 324.9, 36 m high, constructed in 1918), Copco 2 (RKM 324.4, 7.5 m high, constructed in 1925), and Iron Gate (RKM 310.8, 50 m high, constructed in 1962). This project provided access to over 640 km of mainstem and tributary habitat combined that has been inaccessible to anadromous fish for over 100 years^36^.

Here, we examine how large-scale reconnection altered the spatial distribution of Chinook salmon in the Klamath River Basin. The purpose of our study is to provide a quantitative, post-removal assessment of migratory fish responses to the Klamath River dam removals. We used stationary high-resolution multibeam imaging SONAR to enumerate salmonid passage into newly reopened habitats. We documented repeated passage of Chinook salmon beyond the former dam sites during the first two annual migrations into the reconnected river network. We compare these early observations of recolonization with the historical distribution of Chinook salmon and establish a benchmark for post-dam-removal recolonization and recovery in the Klamath Basin. In this paper, we use the term “recolonization” to describe movement into and use of habitat following reconnection. More broadly, this study provides a case study of how restoring connectivity can influence population recovery and ecological resilience in fragmented ecosystems.

## Results

**Fig. 1:**
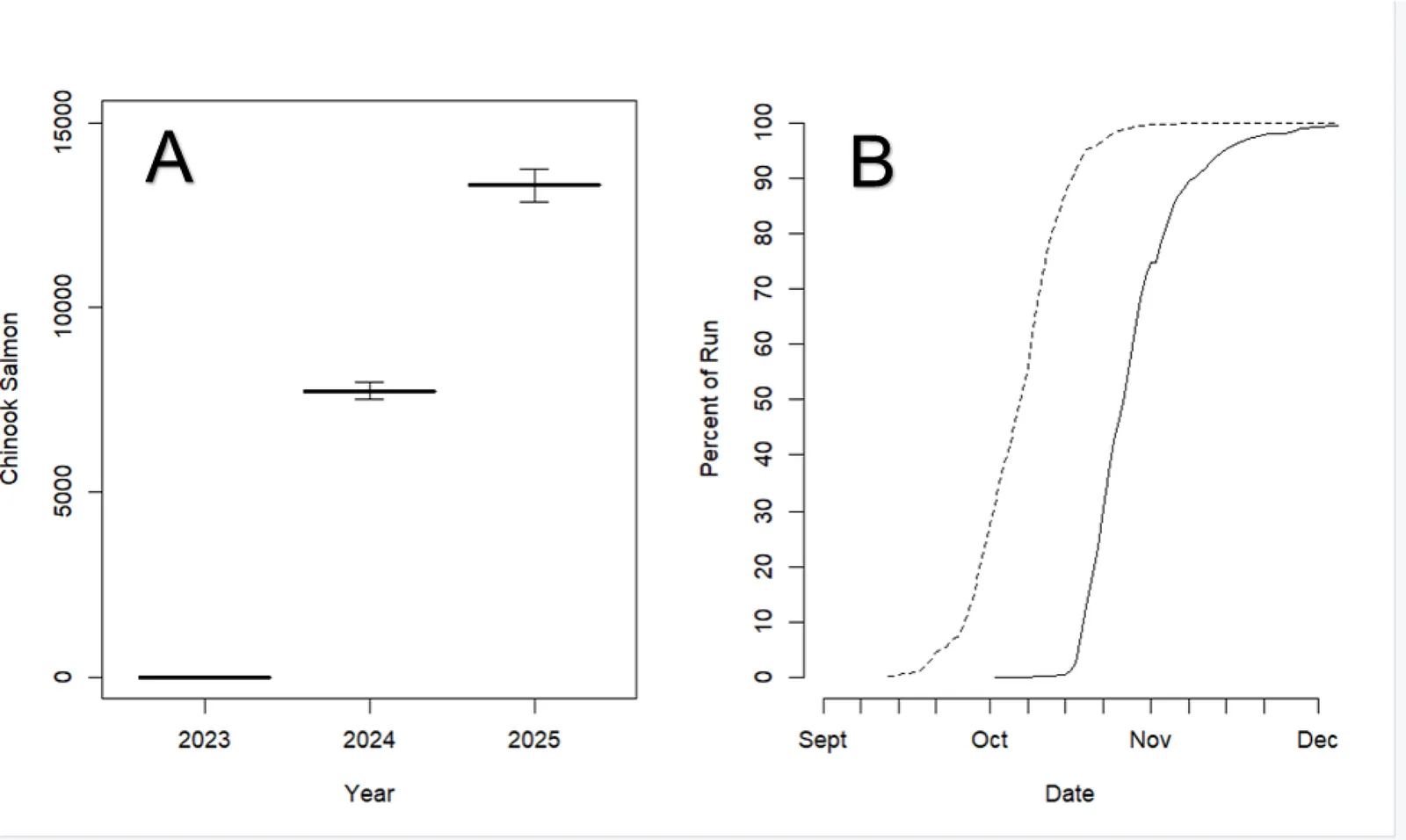
Annual Chinook salmon abundance estimates and run-timing at the former site of Iron Gate Dam by year. (**A**) In 2023 no fish could pass Iron Gate Dam and after removal passage estimates and 95% confidence intervals were estimated using SONAR and species apportionment analyses. (**B**) Cumulative distribution plot of the percentage of the Chinook salmon run with the 2025 run occurring (dashed line) earlier than in 2024 (solid).

Klamath River dam removal concluded on 2-Oct-2024. In 2024, an estimated 7,742 (95% CI: 7,702–7,778) Chinook salmon migrated upstream of the furthest downstream former dam location (Fig. 1A), accounting for over 18% of the total reported Chinook salmon return to the entire Klamath River basin (*n* = 43,654). In 2025, an estimated 13,310 (95% CI: 12,876−13,733) Chinook salmon migrated to habitats upstream of the furthest downstream former dam site, accounting for 19% of the Chinook salmon returning to the Klamath (*n* = 69,852). Return timing varied between years. We detected the first Chinook salmon migrating past the former dam site on 2-Oct-2024 and 12-Sept-2025. The arrival of migrating Chinook salmon in 2024 was tempered for several weeks before accelerating in mid-October and tapering off near the end of December. The earlier arrival of migrating Chinook salmon in 2025 persisted throughout the entire migration season, increasing in early October and largely concluding by early November (Fig. 1B). Median estimated Chinook salmon migration date varied by 18 days between years (25-Oct-2024 and 7-Oct-2025). Maximum estimated daily passage in 2024 was 541 Chinook salmon (21-Oct-2024) and 967 in 2025 (9-Oct-2025).

**Fig. 2:**
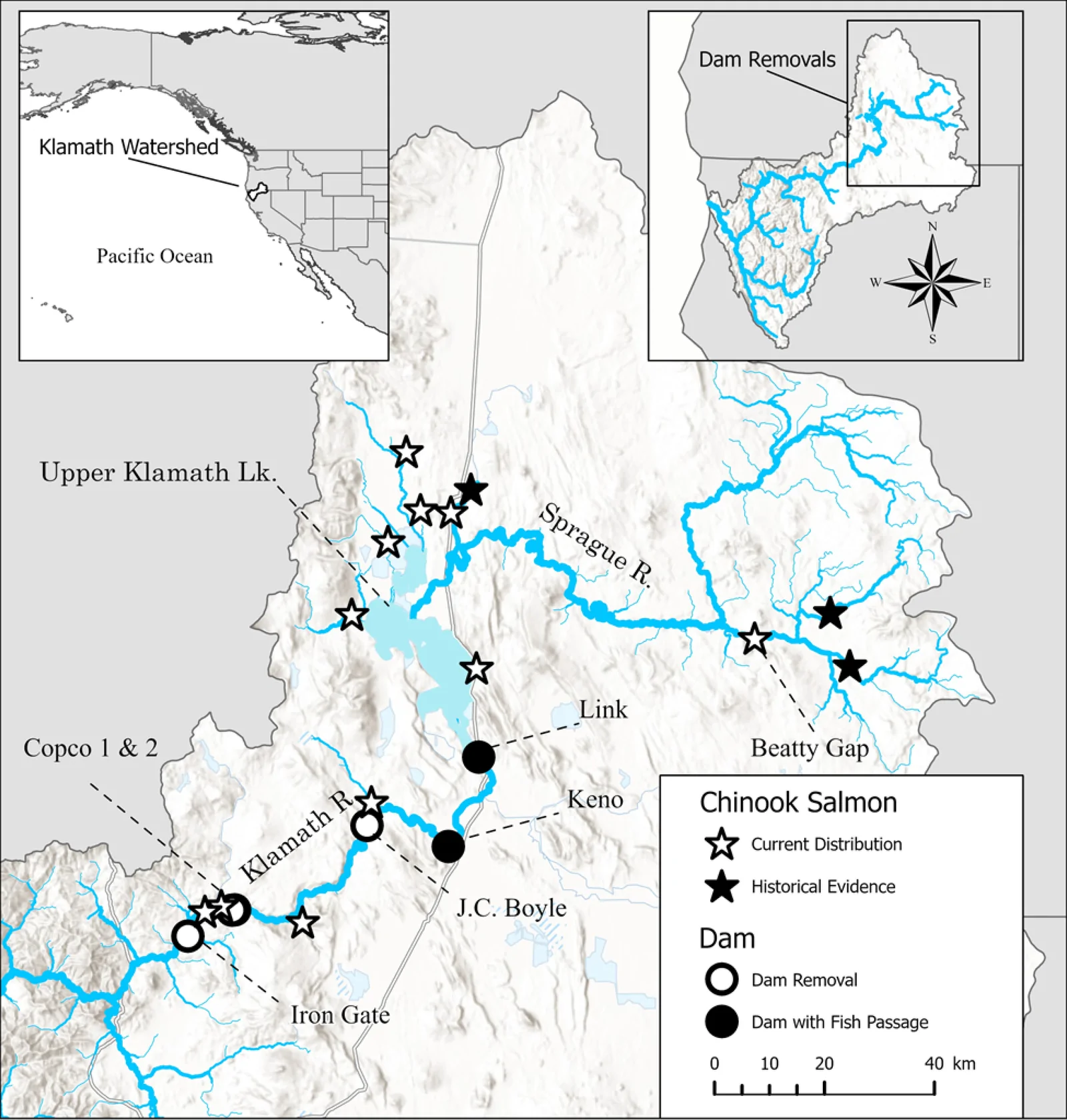
Map of Klamath River dam removals, existing dams with fishways, historical evidence for the upstream extent of Chinook salmon documented before dam construction, and current distribution documented following dam removal in 2024 and 2025. Historical observations are shown only where they extend upstream of the current documented distribution. The map was created by the authors using ArcGIS Pro 3.7.0 (Esri, Redlands, California, USA; https://www.esri.com) and the Esri World Hillshade basemap. Basemap credits: Esri, CGIAR and USGS. No previously published figure was reproduced or adapted.

Within the first two migratory seasons following reconnection, Chinook salmon reoccupied 88% of their documented historical distribution, estimated as the proportion of river length with observations since dam removal relative to river length where fish were observed before dams were constructed (Fig. 2). This expansion encompassed 345 km of the Klamath River, Link River, and 11 tributaries combined. Reoccupation required volitional passage through hydraulically complex and anthropogenically modified corridors, including Class IV+ whitewater gorges, the 7.6-m-tall Keno Dam fishway, the 4.6-m-tall Link River Dam fishway, and transit across Upper Klamath Lake (25,000 ha; ~40 km long × 13 km wide). Chinook salmon were seen at Beatty Gap on the Sprague River, marking the highest current distributional record at an elevation of 1,250 m and the farthest from the Pacific Ocean in the Klamath Basin at over 580 km upstream. Additional anadromous species, including coho salmon (*O. kisutch*), steelhead (*O. mykiss*), and Pacific lamprey (*Entosphenus tridentatus*), were documented entering newly accessible habitats during this period, although their spatial extent was much smaller than that of Chinook salmon.

## Discussion

Here, we provide the first quantitative, post-removal assessment of Chinook salmon recolonization following the world’s largest dam removal. Using high-resolution imaging SONAR, we estimated the abundance and migratory timing of Chinook salmon and used visual surveys to describe their spatial extent into newly reconnected habitats. Re-entry by anadromous fishes was immediate: multiple migratory species, including Chinook salmon, coho salmon, *O. mykiss*, and Pacific lamprey, accessed newly opened reaches within the first migratory season. However, for species other than Chinook salmon, sufficient data are not yet available to produce reliable abundance and migratory timing estimates. Chinook salmon returned to 88% of the documented historical extent prior to dam construction. Relative to other published accounts of fish response to dam removal, including the Elwha River, recolonization was unusually rapid and extensive^19^. Our results demonstrate that Chinook salmon were able to pass all four former dam sites within less than two years after dam removal. We see these results as an encouraging early indicator of success, while emphasizing that longer-term evaluation will require evidence of sustained population performance, particularly successful reproduction and broader ecosystem response.

Many biological and ecological mechanisms led to the positive and immediate response observed in this study. Salmonids combine strong natal philopatry with adaptive dispersal (straying), an evolved bet-hedging strategy that enables exploration of, and establishment in, new habitats^46,47^. Yet the mechanisms that drive straying are complex and remain incompletely understood, limiting our ability to predict how major environmental changes, such as dam removal, will alter its expression^9,48^. Physiological cues can include learned odors of rocks, soil, plants and other chemical constituents or conspecifics in the newly opened habitats^9,49^. Environmental cues for expansion can be related to environmental conditions in the new habitats used such as stream temperature, water quality, and flow conditions^10,50,51^. Once in new locations, salmonids can quickly adapt to their new environment. Physical landscape attributes, for example, such as steeper sections of a river basin that select out for specific phenological or genetic attributes can lead to the success of certain species or life-history strategies of salmonids within a colonizing population^52^. Regardless of the mechanism, these explorations allow salmonids to redistribute and expand their distribution into large portions of a landscape through the dynamic geologic history of the Pacific Rim of Fire^53–55^.

Large-scale dam removal can be an impactful restorative action for both ecological and anadromous fish recovery. Conceptually, ecosystem level responses to dam removal are controlled by multiple causal pathways and interdomain links (i.e. above the dam(s), the former reservoir area(s), and below the dam(s)), which interact to strengthen or dampen any observed or measured set of responses^20^. These responses are dynamic and nonlinear and can be controlled by local or regional factors, making generalizations regarding dam removal more challenging^20^. However, we have now seen several large-scale dam removals that have parallels, irrespective of their local or regional differences. Two dams were removed on the Penobscot River, Maine, U.S.A. in 2012 and 2013 to substantially improve upstream access for migratory fish^56^. Species assemblages have changed from lacustrine to riverine and anadromous, and those riverine and anadromous fishes have increased, in some cases over 400% since dam removal^57^. The Elwha River, Washington, U.S.A. has seen an abundance increase of ~ 500% since dam removal for *O. mykiss* and a re-emergence of key life history strategies that add value to not only abundance but distribution, spatial structure, and diversity^19^.

The Klamath has the potential for an even larger-scale abundance and habitat diversity trajectory. When compared with other large-scale dam removals, a greater percentage of newly opened habitat was recolonized within the first two years^19,57^. One possible factor facilitating this rapid response is that the Klamath River Basin is, in some respects, an “inverted” river basin where the headwaters are broad, lake-filled, and lower gradient streams, leading to larger proportions of the upstream river basin favorable to anadromous salmonids; while as the river moves downstream it cuts through mountains, creating a system that functions opposite to a typical, simple mountain-to-valley river^58^. In addition, newly opened habitats include large spring-fed rivers that provide continuous water quality suitable for cold-water fishes regardless of water-year type or time of year. Together, these features will likely continue to accelerate recolonization and will have long-term implications for ecosystem response relative to steeper, more topographically constrained systems with less predictable streamflow regimes.

Reconnection of the upper Klamath Basin creates the potential for re-emergence of the spring-run Chinook salmon ecotype. The Chinook salmon observed in this study were likely fall-run fish based on autumn migration timing. Spring-run Chinook salmon were historically abundant in the upper Klamath Basin but have not been observed in the vicinity of Iron Gate Dam since the 1970s^44^. Field surveys and recent genetic analyses have documented spring-run populations elsewhere in the watershed. The nearest persistent population occurs in the Salmon River, approximately 200 river kilometers downstream of Iron Gate Dam, and may serve as a genetic reservoir for revitalizing the ecotype in the upper Klamath Basin^44,45^. The reconnected streams upstream of the former dam sites include cold-water habitats and spring-fed rivers that could again support over-summer holding, a key ecological requirement for the spring-run ecotype. Restored connectivity may therefore create conditions under which spring-run expression could reappear over time or support successful reintroduction. Determining the future of spring-run Chinook salmon above former barriers will require dedicated monitoring and genetic study during the spring and summer migration seasons.

Most large-scale dam removals to date have involved diadromous populations with a hatchery component, and a proportion of the fish that recolonized newly accessible habitats were of hatchery origin^19,56^ and the Klamath River is no exception. The initial Chinook salmon source population was, in part, a hatchery population located at the base of Iron Gate Dam, the starting point for both returning and recolonizing the upper watershed beyond the former dams. The Iron Gate Hatchery closed and was partially deconstructed during dam removal. Thus, fish that moved upstream of the former dam sites, like those in the Penobscot River and Elwha River were a mix of natural spawners and returning hatchery fish. However, in all cases fish were able to move past the former or current hatcheries and spawn in newly opened habitats, contributing to the overall population change through more natural processes^19,50^.

Prior to dam removal, high densities of instream spawning occurred immediately downstream of Iron Gate Dam. Following dam removal, this river section experienced elevated fine sediment deposits, reducing habitat quality and promoting upstream movement of natural-origin spawners seeking improved spawning conditions. Regardless of origin, the Chinook salmon that repopulated the upper Klamath River did so volitionally. Their successful passage of historical barriers, rapid expansion of spatial distribution and extensive use of newly available habitats underscore the capacity of Chinook salmon populations of various origins to rapidly respond to large-scale connectivity restoration.

We utilized both rigorous monitoring and technology to help quantify the ecological response to dam removal. The use of SONAR facilitated high-quality and near-continuous data collection through physical challenges including a migrating stream channel, low water clarity, and variable streamflow. Complementary and proximal fish surveys were critical to our SONAR assessment including species composition netting, video monitoring in nearby clear tributaries, and redd-carcass surveys, allowing us to estimate the returning number of Chinook salmon, the variation associated with the estimate, and the distributional extent of recolonization. Artificial intelligence (AI) is starting to aid enumeration of SONAR data, which could streamline the temporal processing of such information. Our SONAR effort is adapted from what was successful in the Elwha River over the last 15 years^19^. This approach provides a scalable framework for monitoring large-scale reconnection projects globally, particularly in large systems where turbidity and channel instability preclude traditional methods.

As large dam removals continue globally, the Klamath River provides a rare quantitative empirical benchmark for how rapidly migratory fishes can recolonize reconnected landscapes when habitat availability and monitoring align. While much has been accomplished with respect to the Klamath River dam removal and the enumeration of the subsequent Chinook salmon response, much more is likely to occur in the Klamath as we have seen from other large-scale dam removals^19,55^. We are in the second year of monitoring Chinook salmon returns. Chinook salmon in the Klamath are primarily two- to four-year-olds, so there are still several more years to understand the response of the first generation of spawning Chinook salmon and the associated ecological responses. Thus, continued monitoring in the Klamath River, like other efforts such as the Elwha River, will be needed to quantify the fish response as the mitigation hatchery contribution declines and stocks shift to wild production. Like other dam removals, ecological responses to the addition of marine-derived nutrients and shifts in the food web due to anadromy will also unfold^59^. Further, it will be important to learn from how the Chinook salmon population responds to a range of climatic, oceanic and hydrologic cycles. Additional monitoring will be necessary to elucidate the responses of other anadromous fishes in the Klamath River, particularly coho salmon, *O. mykiss*, and Pacific lamprey, whose recolonization dynamics, spatial extent, and population trajectories may differ from those observed for Chinook salmon. Further, we note that estimating abundance for other recolonizing fish remains a priority for future work with additional data including extended SONAR deployment to capture complete spawning and migration periods for those species. These data will support not only the future management of the Klamath River but will add to a growing number of connectivity projects and act as a model to establish expectations for future projects worldwide.

## Methods

### SONAR field methods and data review

The SONAR site on the Klamath River was located at RKM 310.7 (41.9319, -122.4403). This site was approximately 100 m downstream from the base of the former Iron Gate Dam which was the artificial upper extent of anadromy from the time of the dam’s construction in the early 1960s to its removal in 2024 (Fig. 2). The site was selected for its proximity to the former dam site as well as fitting three basic criteria for acoustic imaging: (1) narrow enough to accommodate the effective range of the SONAR, (2) the ability to monitor the entire cross section of the river (i.e. no blind spots across the channel), and (3) fish generally move in an upstream direction with little to no milling behavior.

We deployed a stationary ARIS (Adaptive Resolution Imaging Sonar, Explorer models 1200 and 1800, Sound Metrics, Bellevue, WA) to record fish passage events in 2024 and 2025. In 2024, we recorded near-continuous data from 1-Oct to 24-Dec and in 2025 from 12-Sept to 4-Dec. This monitoring encompassed the fall-run Chinook salmon migration period each year. Data gaps totaled 84 h or approximately 2% of the total recording time; therefore, we did not attempt to infill missing data.

ARIS data were processed with ARIS Fish software (Ver. 2.8). Data review followed standard methods for identifying, enumerating, and measuring fish in multi-beam SONAR imagery^19^. In summary, data were transformed using a two-step process that enabled empty periods of time to be efficiently reviewed, while highlighting fish passage events for closer inspection. To isolate moving targets from static features and ambient noise, we applied background subtraction to SONAR data using the default parameters. Then each file was transformed into an echogram using the default parameters. An echogram is a graph of data with distance from the SONAR head on the y-axis and time on the x-axis and enables a reviewer to efficiently review data for moving targets. Potential targets were then evaluated individually to confirm fish-like movement consistent with subcarangiform swimming, measured, and included in the final dataset.

### SONAR data analysis

#### Known-species length and migratory timing data

To parameterize species-specific escapement estimates we compiled known-species fish length and migratory timing data collected in the vicinity of the SONAR site. During the study period Oregon and California Departments of Fish and Wildlife, U.S. Fish and Wildlife Service, Yurok Tribe, Karuk Tribe, and CalTrout captured fish during various adult salmonid monitoring projects in areas upstream of and immediately downstream of the SONAR site. Field measured Chinook salmon averaged 68 cm TL (*n* = 4,185), coho salmon averaged 66 cm TL (*n* = 51) and *O. mykiss* were smaller and averaged 47 cm TL (*n* = 16) (Supplemental A). Regardless of species, overall field measured lengths averaged 67 cm (*n* = 4,252) and were within a cm of SONAR-derived lengths 68 cm (*n* = 15,791). Sample size was determined by the number of fish encountered during field monitoring and the duration of SONAR/video review, not by a priori power analysis.

We estimated Chinook salmon and coho salmon passage timing using underwater video data collected near the SONAR site. Video cameras were placed at tributaries with sufficient water clarity for species identification including Bogus Creek (400 m downstream of SONAR site), Jenny Creek (7 km upstream) and Shovel Creek (27 km upstream). Video cameras were co-located with weirs that directed upstream migrating fish within focal distance of cameras. Chinook salmon median migration date was 26-Oct-2024 (*n* = 1,994 IQR = 13) and 15-Oct-2025 (*n* = 1,498, IQR = 9) (Supplemental B). Coho salmon migrated later in both years, with a median migration date of 14-Dec-2024 (*n* = 94, IQR = 13) and 2-Dec-2025 (*n* = 41, IQR = 18).

#### Modeling species-specific escapement

All statistical modeling was conducted using Bayesian methods coded using BUGS language. To estimate species-specific abundances from SONAR counts, we took advantage of variation in the arrival timing and size among salmonid species from the data described above. This was accomplished by fitting binomial generalized linear models, each with logit link functions, to first estimate the probability of each SONAR counted fish being *O. mykiss* relative to a salmon based on size, and then conditional on this probability, the probability a salmon was a Chinook salmon relative to a coho salmon based on arrival time. More specifically, we defined π as the probability of an observed fish (i) being *O. mykiss*, with logit(π_i_) = β_0_ + β_1_*size_i_ where size was measured in cm, and standard regression parameters βs. We next defined, conditional on being a salmon, Ω as the probability a salmon was a Chinook salmon, with logit(Ω_i_) = α_0_ + α_1_*day_i_ where day was Julian day and standard regression parameters αs. Given these definitions, π remains the probability of an observed fish being *O. mykiss*, (1- π) Ω the probability of an observed fish being a Chinook salmon, and (1- π)(1- Ω) the probability of an observed fish being a coho salmon. For model fitting, both sizes and Julian days within each year were scaled to have mean = 0 and variance = 1.

#### Estimating species-apportioned abundances

To translate the counts of fish obtained from SONAR footage into Chinook salmon abundance estimates, we relied on the statistical modeling results to create an abundance calculating function. The function inputs included the length of the fish as measured by the SONAR unit and the date of observation. The SONAR unit also allows observers to note if the fish is swimming upstream or downstream. To estimate the abundance of salmon remaining upstream of the SONAR unit, we generated separate abundance estimates for each directionality and subtracted the downstream estimates from the upstream estimates. All computations were done across the variation in posterior distribution samples for each parameter to ensure that we properly propagated estimation error through to the final estimates. The size and arrival covariates proved incredibly strong predictors of species which led to very precise parameter estimates and low prediction uncertainty.

In 2024, all SONAR data were reviewed and in 2025, we subsampled SONAR data review at a rate of 20 min h^− 1^, and the full 60 min were reviewed for 13% of the data. This required the statistical abundance model to expand the subsampled counts, and account for the associated sampling error for hours not fully reviewed. This was accomplished via modeling arrivals with a Poisson process with estimated mean parameters representing per-minute expected counts that varied by hour. This allowed the hours with full-footage reviews to fully inform the per-minute mean parameter estimates, and hours with subsampled footage reviews to account for this sampling variability in estimates of the per-minute Poisson counts. Hence, the total abundance estimation process for the 2025 SONAR data relied on two estimation steps. The first step involved estimating the number of Chinook salmon remaining upstream of the SONAR unit based on fish sizes and arrival dates from reviewed footage, which followed the methods described above. Next, the posterior distribution information from the first step was included in the Bayesian statistical model that estimated the total abundance of Chinook salmon in each hour based on per-minute abundance rates.

We constructed likelihoods and specified prior distributions using BUGS language and called the R package nimble^60^ from R^61^ to use Markov chain Monte Carlo (MCMC) simulation to draw samples from the joint posterior distribution of the parameters. For all regression coefficients associated with species-specific abundance estimation, we specified uninformative mean-zero Gaussian priors with precision (variance^–1^) values equaling 0.0001. For all estimated per-minute rate parameters, we specified uninformative mean-zero Gaussian priors with precision 0.01 on the scale of the linear predictor (log link function). We ran three simultaneous MCMC chains and retained 3,000 samples per chain after burn-in sufficient to achieve clear convergence for parameter posterior distributions in each year. Convergence was assessed visually from the trace plots of each MCMC chain and quantitatively by ensuring all Rhat statistics were less than 1.1^62^. For all statistical results related to the Chinook salmon abundance estimates generated with generalized linear models and parameters estimated using Bayesian methods, all MCMC simulations from posterior distributions achieved convergence based on visual assessment of chains showing no trends and clearly well-mixed samples of each posterior distribution, and Rhat values near 1.0.

Abundance estimates in this study were compared against fall-run Chinook salmon abundance estimates compiled for the Klamath River Basin. These estimates were derived from a variety of sources and compiled in the 2024 and 2025 Pacific Fishery Management Council’s review of ocean salmon fisheries^63,64^.

#### Current distribution and historical range

Following basin reconnection, state, federal, and Tribal partners conducted coordinated ground surveys to locate Chinook salmon and quantify the spatial extent of their distribution in newly accessible habitats. Surveys completed during autumn 2024 and 2025 spanned 390 river kilometers of mainstem and tributary habitat combined, including the Klamath River, Link River, and 11 additional tributaries. Site selection was informed by reconstructed historical extent^39,40^, habitat potential identified through state reintroduction planning efforts and verified public observations. Evidence of volitional upstream movement, active spawning, and carcass presence confirmed rapid recolonization during the first migratory seasons following dam removal. Observed distribution was evaluated against historical extent reconstructions to quantify the upstream limit of recolonization relative to the former limit of anadromy. We estimated percent recolonization as the river length currently occupied within the historically documented extent (measured from the upstream-most historical observation downstream to Iron Gate Dam) divided by the total river length with documented historical evidence.

Live fish were collected as part of the broader SONAR monitoring program conducted in coordination with state, federal, and Tribal fisheries representatives. Fish collections were approved by the California Department of Fish and Wildlife under Scientific Collecting Permit No. S-212230001-23030-001-02 and authorized by the National Oceanic and Atmospheric Administration under the Authorizations and Permits for Protected Species State 4(d) Rule for Research Programs, Permit Nos. 28397 and 29249. All collections were performed in accordance with applicable state, federal, and Tribal guidelines and regulations. Reporting followed ARRIVE 2.0 guidelines where applicable to this field-based observational study; reporting items related to controlled treatment allocation, laboratory housing, husbandry, and humane endpoints were not applicable to passive SONAR monitoring, video monitoring, or associated field collections.

## Electronic Supplementary Material

Below is the link to the electronic supplementary material.


Supplementary Material 1



Supplementary Material 2


## Data Availability

The datasets generated and analyzed during the current study are available in the Zenodo repository, DOI: https://doi.org/10.5281/zenodo.21458527.

## Code availability

The R code used for species apportionment and abundance estimation is available in the Zenodo repository, DOI: https://doi.org/10.5281/zenodo.21458527.

## References

[1] Liu, J. et al. How does habitat fragmentation affect the biodiversity and ecosystem functioning relationship? *Landsc. Ecol.* **33**, 341–352 (2018).

[2] Edge, C. B. et al. Habitat alteration and habitat fragmentation differentially affect beta diversity of stream fish communities. *Landsc. Ecol.* **32**, 647–662 (2017).

[3] Di Giulio, M., Holderegger, R. & Tobias, S. Effects of habitat and landscape fragmentation on humans and biodiversity in densely populated landscapes. *J. Environ. Manage.* **90**, 2959–2968 (2009). 19493609 10.1016/j.jenvman.2009.05.002

[4] Cameron, D. R., Schloss, C. A., Theobald, D. M. & Morrison, S. A. A framework to select strategies for conserving and restoring habitat connectivity in complex landscapes. *Conserv. Sci. Pract.* **4**, e12698 (2022).

[5] Olin, A. B. et al. Predation and spatial connectivity interact to shape ecosystem resilience to an ongoing regime shift. *Nat. Commun.* **15**, 1304 (2024). 38347008 10.1038/s41467-024-45713-1 PMC10861472

[6] Wang, M. et al. Habitat connectivity drives panda recovery. *Curr. Biol.* **34**, 3894–3904 (2024). 39127049 10.1016/j.cub.2024.07.037

[7] Pimm, S. L., Willigan, E., Kolarova, A. & Huang, R. Reconnecting nature. *Curr. Biol.* **31**, R1159–R1164 (2021). 34637722 10.1016/j.cub.2021.07.040

[8] Lotze, H. K., Coll, M., Magera, A. M., Ward-Paige, C. & Airoldi, L. Recovery of marine animal populations and ecosystems. *Trends Ecol. Evol.* **26**, 595–605 (2011). 21852017 10.1016/j.tree.2011.07.008

[9] Pess, G. R., Quinn, T. P., Gephard, S. R. & Saunders, R. Re-colonization of Atlantic and Pacific rivers by anadromous fishes: linkages between life history and the benefits of barrier removal. *Rev. Fish. Biol. Fish.* **24**, 881–900 (2014).

[10] Pess, G. R., Hilborn, R., Kloehn, K. & Quinn, T. P. The influence of population dynamics and environmental conditions on pink salmon (*Oncorhynchus gorbuscha*) recolonization after barrier removal in the Fraser River, British Columbia, Canada. *Can. J. Fish. Aquat. Sci.* **69**, 970–982 (2012).

[11] Ripple, W. J., Beschta, R. L., Fortin, J. K. & Robbins, C. T. Trophic cascades from wolves to grizzly bears in Yellowstone. *J. Anim. Ecol.* **83**, 223–233 (2014). 24033136 10.1111/1365-2656.12123

[12] Pringle, R. M. Upgrading protected areas to conserve wild biodiversity. *Nature* **546**, 91–99 (2017). 28569807 10.1038/nature22902

[13] Preston, J. et al. Seascape connectivity: evidence, knowledge gaps and implications for temperate coastal ecosystem restoration practice and policy. *npj Ocean. Sustain.* **4**, 33 (2025). 40520358 10.1038/s44183-025-00128-3 PMC12162346

[14] Beechie, T. J. et al. Process-based principles for restoring river ecosystems. *BioScience* **60**, 209–222 (2010).

[15] Munsch, S. H. et al. Dam removal enables diverse juvenile life histories to emerge in threatened salmonids repopulating a heterogeneous landscape. *Front. Ecol. Evol.* **11**, 1188921 (2023).

[16] Keel, D. J. et al. A molecular specimen bank for contemporary and future study captures landscape-scale biodiversity baselines before Klamath River dam removal. *Sci. Rep.* **15**, 20679 (2025). 40596150 10.1038/s41598-025-07042-1 PMC12217707

[17] Ward, J. V. The four-dimensional nature of lotic ecosystems. *J. N Am. Benthol Soc.* **8**, 2–8 (1989).

[18] Mason, R. J. et al. Rebalancing river lateral connectivity: an interdisciplinary focus for research and management. *WIREs Water*. **12**, e1766 (2025).

[19] Pess, G. R. et al. Initial responses of Chinook salmon (*Oncorhynchus tshawytscha*) and *O. mykiss* (*Oncorhynchus mykiss*) to removal of two dams on the Elwha River, Washington State, USA. *Front. Ecol. Evol.* **12**, 1241028 (2024).

[20] Bellmore, J. R. et al. Conceptualizing ecological responses to dam removal: if you remove it, what’s to come? *BioScience* **69**, 26–39 (2019). 30647476 10.1093/biosci/biy152 PMC6327834

[21] Magilligan, F. J. et al. River restoration by dam removal: enhancing connectivity at watershed scales. *Elementa* **4**, 000108 (2016).

[22] McCaffery, R. M., Duda, J. J., Soissons, L., & Roussel, J. Editorial: Large-scale dam removal and ecosystem restoration. *Front. Ecol. Evol.* **12**, 1471146 (2024).

[23] Ritchie, A. C. et al. Morphodynamic evolution following sediment release from the world’s largest dam removal. *Sci. Rep.* **8**, 13279 (2018). 30185796 10.1038/s41598-018-30817-8 PMC6125403

[24] Wohl, E. & Scott, D. N. Wood and sediment storage and dynamics in river corridors. *Earth Surf. Process. Landf.* **42**, 5–23 (2017).

[25] Olden, J. D. Challenges and opportunities for fish conservation in dam impacted waters. In *Conservation of Freshwater Fishes* (eds Closs, G. P., Krkosek, M. & Olden, J. D.) 107–142 (Cambridge Univ. Press, 2016).

[26] Hitt, N. P., Eyler, S. & Wofford, J. E. Dam removal increases American eel abundance in distant headwater streams. *Trans. Am. Fish. Soc.* **141**, 1171–1179 (2012).

[27] Hogg, R. S., Coghlan, S. M., Jr, Zydlewski, J. & Gardner, C. Fish community response to a small-stream dam removal in a Maine coastal river tributary. *Trans. Am. Fish. Soc.* **144**, 467–479 (2015).

[28] Fraik, A. K. et al. The impacts of dam construction and removal on the genetics of recovering *O. mykiss* (*Oncorhynchus mykiss*) populations across the Elwha River watershed. *Genes* **12**, 89 (2021). 33450806 10.3390/genes12010089 PMC7828262

[29] Duda, J. J. et al. Reconnecting the Elwha River: spatial patterns of fish response to dam removal. *Front. Ecol. Evol.* **9**, 765488 (2021).

[30] Cordoleani, F. et al. Threatened salmon rely on a rare life history strategy in a warming landscape. *Nat. Clim. Change*. **11**, 982–988 (2021).

[31] Munsch, S. H., Greene, C. M., Mantua, N. J. & Satterthwaite, W. H. One hundred-seventy years of stressors erode salmon fishery climate resilience in California’s warming landscape. *Glob Change Biol.* **28**, 2183–2201 (2022). 10.1111/gcb.16029 35075737

[32] Connors, B. M. et al. Chinook salmon diversity contributes to fishery stability and trade-offs with mixed-stock harvest. *Ecol. Appl.* **32**, e2709 (2022). 36131546 10.1002/eap.2709

[33] National Research Council. *Hydrology, Ecology, and Fishes of the Klamath River Basin* (National Academies, 2008).

[34] National Research Council. *Endangered and Threatened Fishes in the Klamath River Basin: Causes of Decline and Strategies for Recovery* (National Academies, 2004).

[35] Hodge, B. W., Wilzbach, M. A., Duffy, W. G., Quiñones, R. M. & Hobbs, J. A. Life history diversity in Klamath River *O. mykiss*. *Trans. Am. Fish. Soc.* **145**, 227–238 (2016).

[36] Diver, S., Oberholzer Dent, J. R., Sarna-Wojcicki, D., Reed, R. & Dill-De Sa, C. Recasting Klamath dam removal as eco-cultural revitalization and restorative justice through Karuk Tribal leadership. *Water* **16**, 2295 (2024).

[37] Schindler, D. E. et al. Pacific salmon and the ecology of coastal ecosystems. *Front. Ecol. Environ.* **1**, 31–37 (2003).

[38] Sowerwine, J., Mucioki, M., Sarna-Wojcicki, D. & Hillman, L. Reframing food security by and for Native American communities: a case study among tribes in the Klamath River basin of Oregon and California. *Food Secur.* **11**, 579–607 (2019).

[39] Hamilton, J. B., Curtis, G. L., Snedaker, S. M. & White, D. K. Distribution of anadromous fishes in the Upper Klamath River watershed prior to hydropower dams—a synthesis of the historical evidence. *Fisheries* **30**, 10–20 (2005).

[40] Hamilton, J. B. et al. The persistence and characteristics of Chinook salmon migrations to the upper Klamath River prior to exclusion by dams. *Oreg Hist. Q.* **117**, 326–377 (2016).

[41] Stocking, R. W. & Bartholomew, J. L. Spatial and temporal occurrence of the salmonid parasite *Ceratomyxa shasta* in the Oregon–California Klamath River Basin. *J. Aquat. Anim. Health*. **18**, 194–202 (2006).

[42] Fujiwara, M., Mohr, M. S., Greenberg, A., Foott, J. S. & Bartholomew, J. L. Effects of ceratomyxosis on population dynamics of Klamath fall-run Chinook salmon. *Trans. Am. Fish. Soc.* **140**, 1380–1391 (2011).

[43] Prince, D. J. et al. The evolutionary basis of premature migration in Pacific salmon highlights the utility of genomics for informing conservation. *Sci. Adv.* **3**, e1603198 (2017). 28835916 10.1126/sciadv.1603198 PMC5559211

[44] Thompson, T. Q. et al. Anthropogenic habitat alteration leads to rapid loss of adaptive variation in wild salmon populations. *Proc. Natl. Acad. Sci. USA*. **116**, 177–186 (2019). 30514813 10.1073/pnas.1811559115 PMC6320526

[45] Thompson, N. F. et al. A complex phenotype in salmon controlled by a simple change in migratory timing. *Science* **370**, 609–613 (2020). 33122386 10.1126/science.aba9059

[46] Quinn, T. P. & Dittman, A. H. Pacific salmon migrations and homing: mechanisms and adaptive significance. *Trends Ecol. Evol.* **5**, 174–177 (1990). 21232348 10.1016/0169-5347(90)90205-R

[47] Keefer, M. L. & Caudill, C. C. Homing and straying by anadromous salmonids: a review of mechanisms and rates. *Rev. Fish. Biol. Fish.* **24**, 333–368 (2014).

[48] Hendry, A. P., Castric, V., Kinnison, M. T. & Quinn, T. P. The evolution of philopatry and dispersal: homing versus straying in salmonids. In *Evolution Illuminated: Salmon and Their Relatives* (eds Hendry, A. P. & Stearns, S. C.) 52–91 (Oxford Univ. Press, 2004).

[49] Nordeng, H. Is the local orientation of anadromous fishes determined by pheromones? *Nature* **223**, 411–413 (1971). 10.1038/233411a0 16063406

[50] Liermann, M. et al. Relocation and recolonization of coho salmon in two tributaries to the Elwha River: implications for management and monitoring. *Trans. Am. Fish. Soc.* **146**, 955–966 (2017).

[51] Perrier, C., Evanno, G., Belliard, J., Guyomard, R. & Bagliniere, J. L. Natural recolonization of the Seine River by Atlantic salmon (*Salmo salar*) of multiple origins. *Can. J. Fish. Aquat. Sci.* **67**, 1–4 (2010).

[52] Horn, R. L., Caisman, J., Kamphaus, C. & Narum, S. R. Genomic insights into local adaptation and migration success in reintroduced coho salmon of the Wenatchee River basin. *Trans. Am. Fish. Soc.* **154**, 414–423 (2025).

[53] Anderson, J. H. et al. Dispersal and tributary immigration by juvenile coho salmon contribute to spatial expansion during colonization. *Ecol. Freshw. Fish.* **22**, 30–42 (2013).

[54] Sahashi, G., Morita, K. & Kishi, D. Spatial expansion and increased population density of masu salmon parr independent of river restoration. *Ichthyol. Res.* **65**, 496–501 (2018).

[55] Dunmall, K. M. et al. Invading and range-expanding pink salmon inform management actions for marine species on the move. *ICES J. Mar. Sci.* **82**, fsae199 (2025).

[56] Trinko Lake, T., Ravana, K. R. & Saunders, R. Evaluating changes in diadromous species distributions and habitat accessibility following the Penobscot River Restoration Project. *Mar. Coast Fish.* **4**, 284–293 (2012).

[57] Whittum, K. A. et al. Fish assemblages in the Penobscot river: a decade after dam removal. *Mar. Coast Fish.* **15**, e10227 (2023).

[58] Michalak, M. J. et al. Neogene faulting, basin development, and relief generation in the southern Klamath Mountains (USA). *Geosphere* **20**, 237–266 (2024).

[59] Morley, S. A. et al. Shifting food web structure during dam removal—disturbance and recovery during a major restoration action. *PLoS One*. **15**, e0239198 (2020). 32991602 10.1371/journal.pone.0239198 PMC7523948

[60] Goldstein, B. R., Turek, D., Ponisio, L., Zhang, W. & de Valpine, P. nimbleEcology: Distributions for Ecological Models in ‘nimble’. R package version 0.5.0 (2024).

[61] R Core Team. *R: A Language and Environment for Statistical Computing* (R Foundation for Statistical Computing, 2021).

[62] Gelman, A., Hwang, J. & Vehtari, A. Understanding predictive information criteria for Bayesian models. *Stat. Comput.* **24**, 997–1016 (2014).

[63] Pacific Fishery Management Council. *Review of 2024 Ocean Salmon Fisheries, Stock Assessment and Fishery Evaluation Document for the Pacific Coast Salmon Fisheries Management Plan* (Pacific Fishery Management Council, 2025).

[64] Pacific Fishery Management Council. *Review of 2025 Ocean Salmon Fisheries, Stock Assessment and Fishery Evaluation Document for the Pacific Coast Salmon Fisheries Management Plan* (Pacific Fishery Management Council, 2026).

